# RNA-seq based transcriptional analysis of *Saccharomyces cerevisiae* and *Lachancea thermotolerans* in mixed-culture fermentations under anaerobic conditions

**DOI:** 10.1186/s12864-019-5511-x

**Published:** 2019-02-18

**Authors:** Kirti Shekhawat, Hugh Patterton, Florian F. Bauer, Mathabatha E. Setati

**Affiliations:** 1 Institute for Wine Biotechnology, Department of Viticulture and Oenology, Stellenbosch University, Stellenbosch, Western Cape South Africa; 2 Centre for Bioinformatics and Computational Biology, Stellenbosch University, Stellenbosch, Western Cape South Africa

**Keywords:** Transcriptome, RNA-seq, Wine fermentation, Yeast interactions, *Lachancea thermotolerans*, *Saccharomyces cerevisiae*, Mixed-cultures, Anoxia

## Abstract

**Background:**

In wine fermentation starter cultures, the blending of non-*Saccharomyces* yeast with *Saccharomyces cerevisiae* to improve the complexity of wine has become common practice, but data regarding the impact of co-cultivation on yeast physiology and on genetic and metabolic regulation remain limited. Here we describe a transcriptomic analysis of mixed fermentations of *Saccharomyces cerevisiae* and *Lachancea thermotolerans*. The fermentations were carried out in carefully controlled environmental conditions in a bioreactor to reduce transcriptomic responses that would be due to factors other than the presence of the second species.

**Results:**

The transcriptomic data revealed that both yeast species showed a clear response to the presence of the other. Affected genes primarily belonged to two groups: genes whose expression can be linked to the competition for certain trace elements such as copper and iron, as well as genes required for cell wall structure and integrity. Furthermore, the data revealed divergent transcriptional responses with regard to carbon metabolism in response to anoxic conditions.

**Conclusions:**

The results suggest that the mixed fermentation created a more competitive and stressful environment for the two species than single strain fermentations independently from total biomass, i.e. competition between cells of the same species is less stressful, or may present a different set of challenges, than interspecies competition. The changes in cell wall and adhesion properties encoding genes suggest that the adjustment of physical contact between cells may play a direct role in the response to the presence of competing species.

**Electronic supplementary material:**

The online version of this article (10.1186/s12864-019-5511-x) contains supplementary material, which is available to authorized users.

## Background

During crushing of grapes, grape juice is exposed to air and becomes saturated with oxygen. Oxygen may also be discretely introduced into the wine fermentation process at various stages in amounts ranging from 2 mg L^− 1^ to 6 mg L^− 1^ depending on the method employed [1, 2]. Most of the oxygen is rapidly consumed by yeast cells, and concomitant CO_2_ production has a negative impact on oxygen dissolution, creating an anaerobic environment [1]. Several yeast species that constitute the wine fermentation community including members of the genera *Saccharomyces*, *Torulaspora*, *Hanseniaspora*, *Lachancea*, *Pichia*, *Candida* and *Starmerella* are facultative anaerobes that can grow and survive under these conditions. However, most of the non-*Saccharomyces* yeasts have high oxygen demands and low fermentation capacity compared to *Saccharomyces* species, particularly *Saccharomyces cerevisiae* [3–5]. Consequently, such species are used in mixed cultures with *S. cerevisiae* to ensure complete conversion of grape sugars to ethanol, CO_2_ and other metabolites that constitute the wine fermentation bouquet. This practice has gained tremendous interest in the global wine industry over the past decade, mainly due to improved wine flavour and aroma complexity, as well as potential reduction in ethanol levels, resulting from the underlying yeast-yeast interactions [6–12]. Consequently, understanding the interaction between *Saccharomyces* and non-*Saccharomyces* yeasts has become a central focus of ecological and of wine-related research. The nature of some of the ecological interactions between two yeast species have been previously evaluated. The data show that in mixed fermentation of *S. cerevisiae* and non-*Saccharomyces* yeasts, *S. cerevisiae* displays antagonistic interaction towards non-*Saccharomyces* yeasts such as *Torulaspora delbrueckii*, *Hanseniaspora guilliermondii*, *Lachancea thermotolerans* and *Kluyveromyces lactis* [13–15]. Early studies revealed that presence of *S. cerevisiae* cells at a high concentration causes cellular death in *T. delbrueckii* and *L. thermotolerans* [13]. These studies were subsequently followed by pioneering work attributing this antagonism to direct cell-cell contact as well as the production of antimicrobial peptides by *S. cerevisiae* [15, 16]. The data strongly suggest the existence of specific physical and metabolic interactions between yeast species, but do not provide any insights about the molecular mechanism behind such interactions, and little is known about the molecular factors influencing the response of any yeast species to the presence of another species. Such studies are challenging because of the complexity of multispecies systems and of ecological interactions. In particular, very few investigations have thus far been published reporting genome-wide data sets for such interactions, and most of these studies have primarily been reporting on the response of *S. cerevisiae* to the presence of another species. For instance, DNA microarray-based transcriptome analyses and mass spectrometry-based proteome analyses have been used to study the interaction between yeast and bacteria as well as between *S. cerevisiae* and non-*Saccharomyces* yeasts under oenological conditions [17–24]. Furthermore, these studies have usually relied on batch fermentation conditions. Such conditions make it difficult to differentiate the relative impact of the continuous changes in media composition from the specific response of one yeast species to the presence of the other.

In the current study, we evaluated the transcriptomic response of *L. thermotolerans* and *S. cerevisiae* in mixed fermentations when compared to single strain cultures in the same environmental conditions. We selected *L. thermotolerans* as a non-*Saccharomyces* wine yeast as that yeast has already been commercialised for use in mixed starter fermentations. Mixed culture fermentation with *L. thermotolerans* are known for leading to enhanced concentration of higher alcohols (particularly 2-phenylethanol), l-lactic acid, glycerol and esters, while in some conditions also resulting in lower ethanol wines [10]. The genome of this yeast has been sequenced and the genome sequence has been partially annotated. As demonstrated in previous studies [3, 5], oxygen enhances the growth and persistence of *L. thermotolerans* in mixed starter fermentations. To better characterise the molecular nature of the interactions, we used a controlled bioreactor system that allowed maintenance of two species in fermentation with continuous in-flow and out-flow of medium. The conditions were set to ensure that the total biomass of mixed and single species fermentations, and the environmental factors that strongly impact gene transcription in fermentative conditions such as nutrient availability, oxygen, ethanol and hexose concentrations, were maintained at similar levels in all fermentations. These settings should restrict the transcriptomic response to factors linked to the presence of a second species.

## Results

### Optimisation of fermentation conditions

**Fig. 1:**
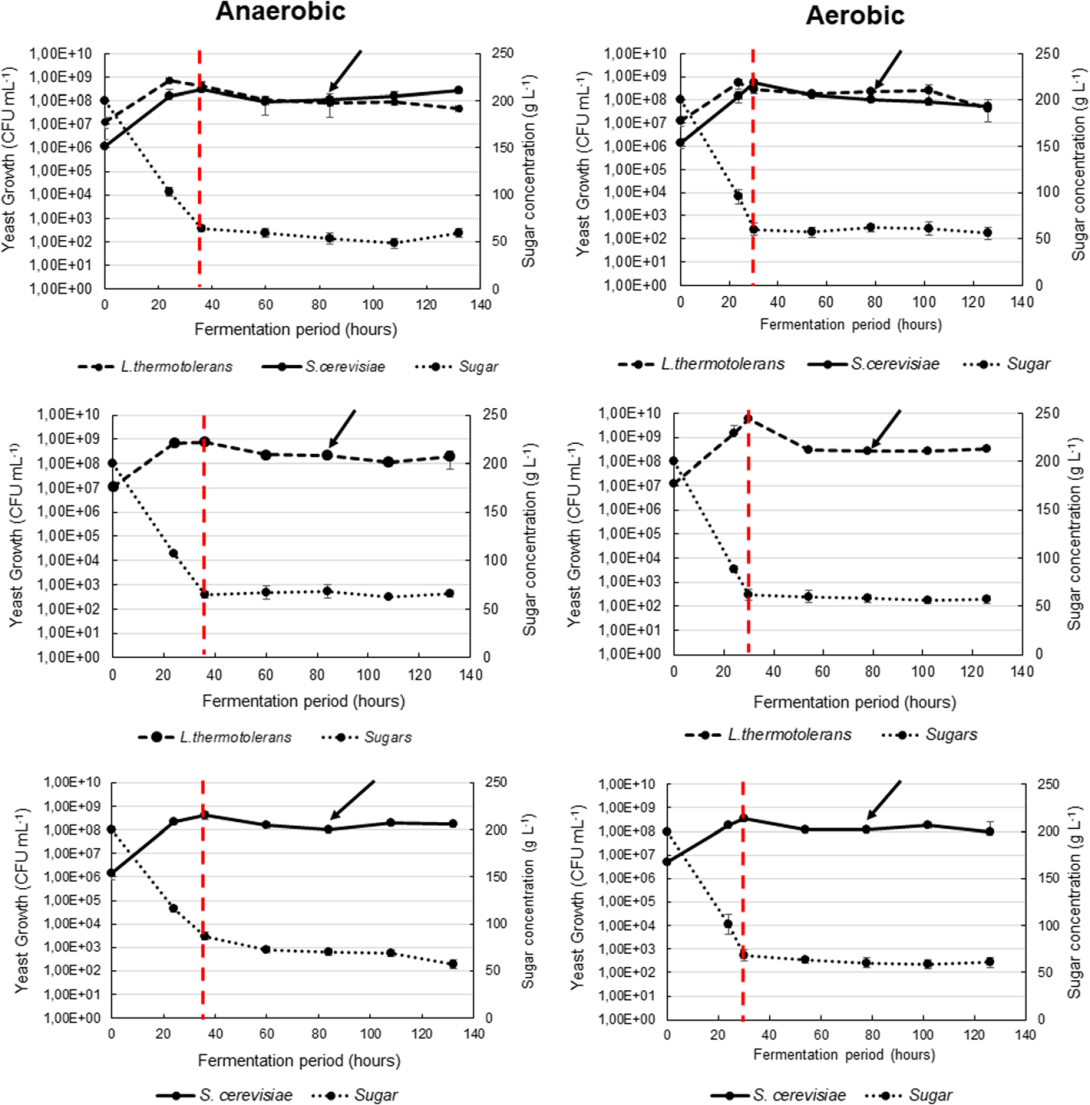
Fermentation kinetics and population dynamics of *S. cerevisiae* and *L. thermotolerans* in single and mixed fermentation under anaerobic (left panel) and aerobic (right panel) fermentation conditions. The red line indicates the start of continuous culture, the arrows indicate the sampling point

Multispecies interaction studies at the molecular, transcriptomic or proteomic level face significant challenges. Indeed, when such studies are carried out in standard batch fermentation conditions, both species continuously modify gene expression to respond to the dynamic environment. Furthermore, population evolution leads to continuous change in the level of mutual exposure. In such conditions, any specific transcriptional response of one species to the presence of the other species will be hidden within a broader transcriptional response to changes in the environment. To overcome this problem, and to focus the investigation on the transcriptomic signature of the interaction between species, a system with similarity to a chemostat was optimised. The specific growth rate of *S. cerevisiae* and *L. thermotolerans* monocultures under anaerobic conditions, was found to be 0.2 h^− 1^ at a dilution rate of 0.1 h^− 1^ and 0.17 h^− 1^ at a dilution rate of 0.075 h^− 1^, respectively, and similar cell concentrations were obtained (Table 1). In order to avoid a washout of *L. thermotolerans* in mixed fermentations, the cultures were co-inoculated and cultivated in batch for 30 h, and then switched to continuous mode at a dilution rate of 0.1 h^− 1^ for the anaerobic fermentation. In contrast, under aerobic conditions, the two yeasts displayed similar specific growth rates and reached comparable cell concentrations under the same dilution rate (Table 1). Consequently, the same dilution rate could be applied in the mixed culture fermentations. The aim of this optimisation was to ensure similar population densities and similar growth medium composition in both monocultures and mixed culture fermentations. As illustrated in Fig. 1, under these optimised conditions, the species display similar growth rates in single and mixed fermentations, and after 48 h of continuous culture, the total number of cells, as well as the sugar concentrations were similar in all the fermentations. The viable counts obtained from samples used for RNA extraction and expression analysis, showed that the cell concentrations of both species were ≈ 10^8^ CFU mL^− 1^ in both mono- and mixed-cultures (Table 1).

**Table 1:** A summary of the dilutions rates applied to maintain similar cell concentrations, as well as the chemical fermentation parameters at the time of sampling for transcriptome analysis

Fermentations	Dilution rate h^− 1^	CFU mL^− 1^ at 48 h	Sugar concentration at 48 h (g L^− 1^)	Glycerol concentration at 48 h (g L^− 1^)	μMax at exponential phase (h^− 1^)
*L. thermotolerans*-AN	0.075	2.1E+ 08	68.0	2.92	0.17
*S. cerevisiae*-AN	0.10	1.2E+ 08	62.5	2.40	0.20
Mixed-AN	0.10	Sc- 1.1E+ 08 Lt- 8.6E+ 07	59.6	3.14	
*S. cerevisiae*-AR	0.125	1.0E+ 08	60.0	1.09	0.23
*L. thermotolerans*-AR	0.125	2.5E+ 08	58.0	1.34	0.24
Mixed-AR	0.125	Sc- 1.1E+ 08 Lt- 2.3E+ 08	62.0	1.12

### Global analysis of the transcriptome

**Fig. 2:**
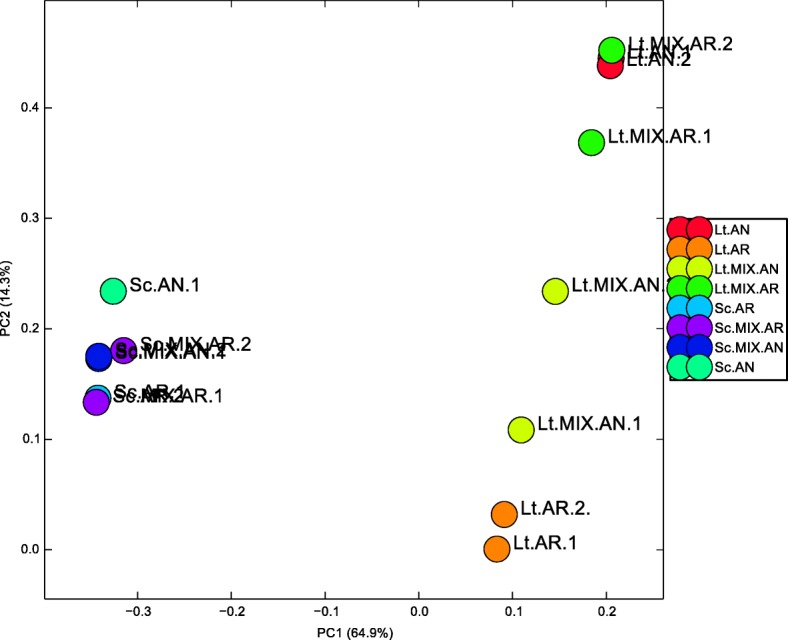
Principal Component Analysis (PCA) plot of the biological replicates of transcripts from *Lachancea thermotolerans* (Lt) and *Saccharomyces cerevisiae* (Sc) monocultures and mixed cultures (Lt.MIX and Sc.MIX) under aerobic (AR) and anaerobic (AN) conditions

The RNA-sequencing was performed for two biological replicates of each fermentation. RNA extractions were performed on samples collected after the two species were in contact for 48 h and had maintained similar cell concentrations (Fig. 1). This was done to allow sufficient metabolic interaction under conditions akin to a steady state. After filtering and trimming, RNAseq samples contained between 11 and 15 Mb total reads (Additional file 1: Table S1). These reads were mapped to the S288CplusLT chimeric genome which was generated after cross-mapping between *S. cerevisiae* and *L. thermotolerans* was found to be less than 1%. The data show that the mixed culture transcriptome had low coverage of *L. thermotolerans*. In particular, the oxygenated fermentation samples generated a total sum of ≈ 2 Mb *L. thermotolerans* reads between the two biological replicates, which is below the recommended threshold. Nevertheless, the data were considered useful for certain analyses. One of the *S. cerevisiae* anaerobic monocultures (Sc.AN.2) appeared to be mixed and was therefore not considered in subsequent analyses. Principal Component Analysis (PCA) performed on normalized TPM data showed that the samples clustered together in a yeast and treatment specific manner, with only minor variations between the independent biological replicates (Fig. 2). *L. thermotolerans* displayed higher levels of transcriptomic change under each condition compared to *S. cerevisiae*, as evident in the separation of the samples along PC2. The statistical analysis was done using Benjamini-Hochberg on all highly-expressed genes to control the FDR.

### Overview of transcriptional response in mixed fermentation

**Fig. 3:**
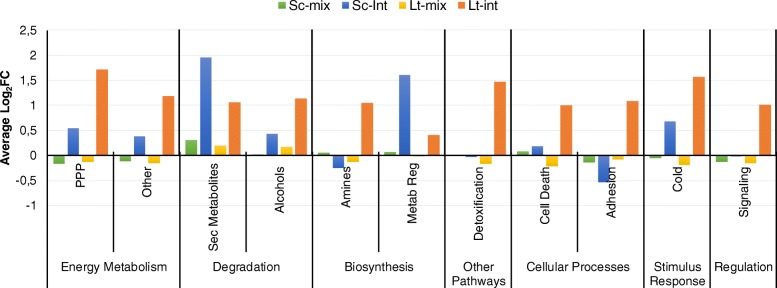
Overall comparison of transcriptional response of *Saccharomyces cerevisiae* (Sc) and *Lachancea thermotolerans* (Lt) to mixed-culture environment (Sc-mix and Lt-mix), and to the combined effect of mixing and anoxia (Sc-int and Lt-int)

The effect of mixing (MIX) on the gene transcription in *S. cerevisiae* and *L. thermotolerans* was assessed by comparing the transcriptome of the mixed cultures to the monocultures under both aerobic and anaerobic conditions (i.e Sc-Lt-AN and Sc-Lt-AR compared to Sc-AN, Sc-AR, Lt-AN and Lt-AR). Genes uniquely differentially expressed in the mixed fermentations compared to monocultures were identified (Additional file 2: Table S2). The genes differentially expressed under anaerobic conditions (AN) were determined by comparing the anaerobic cultures to the aerobic cultures (i.e Sc-Lt-AN, Sc-AN and Lt-AN compared to Sc-Lt-AR, Sc-AR and Lt-AR). Furthermore, common genes differentially expressed in both MIX and AN sets were considered to be affected by the interaction (INT) between mixing and anoxia (Additional file 2: Table S2). For the initial global analysis, the differentially expressed genes (DEGs) were visualized by overlaying the data on the Biocyc Omics dashboard. Overall, the interaction between mixing and anoxia (INT) elicited a stronger response in *L. thermotolerans* than in *S. cerevisiae* (Fig. 3). In *S. cerevisiae*, only the degradation of secondary metabolites as well as the biosynthesis of metabolic regulators were significantly up-regulated. These included *TSL1*, *TPS1*, *TPS2* and *TPS3*, involved in trehalose biosynthesis (Additional file 3: Table S3). Conversely, *L. thermotolerans* displayed an up-regulation of several biological processes most notably detoxification, cell death, adhesion as well as response to stimulus (Fig. 3). The highly up-regulated detoxification and adhesion-related genes in *L. thermotolerans* included *ALD2*, *ALD5*, *SOD1*, *SOD2*, *DLD1*, *CTT1*, and *HSP12*, *FLO9*, *NRG1*, *SDS3* and *CCW12*, respectively (Additional file 3: Table S3).

### Response to anoxia in mixed fermentations

**Fig. 4:**
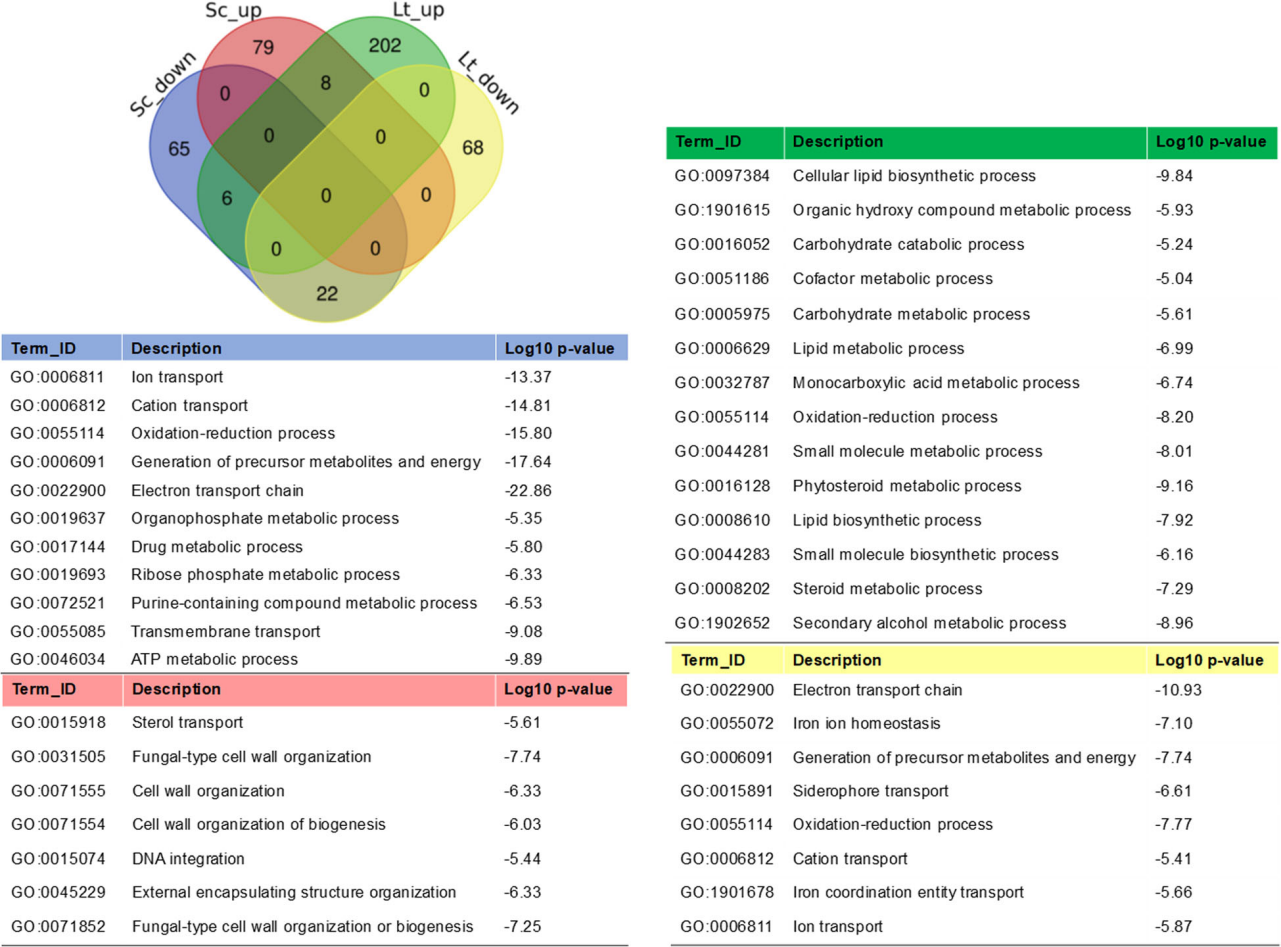
A Venn diagram showing the number of differentially expressed genes (DEGs) commonly regulated in *S. cerevisiae* and *L. thermotolerans* in a mixed-culture fermentation in response to anoxia compared to aerobic mixed-culture. The enriched GO terms reflecting the biological processes associated with the DEGs are presented in the associated tables

The anaerobic and aerobic mixed cultures (Sc-Lt-AN vs Sc-Lt-AR) were compared to assess the transcriptional response of *S. cerevisiae* and *L. thermotolerans* to anoxia in a mixed fermentation setting. Considering DEGs with log_2_FC ≥ 1.5 or ≤ − 1.5, only 8 genes were up-regulated genes and 22 down-regulated genes were common between *S. cerevisiae* and *L. thermotolerans* (Fig. 4). Gene ontology (GO) enrichment showed that cation transport, electron transport chain, generation of precursor metabolites and energy, were some of the highly down-regulated biological processes in both yeasts. By contrast, biological processes enriched in up-regulated genes were distinct between the two yeasts. For instance, in *S. cerevisiae*, sterol transport, cell wall organization and associated processes were enriched while in *L. thermotolerans* oxidation-reduction process, carbohydrate catabolism as well as cellular lipid biosynthetic processes were enriched (Fig. 4).

### Response to mixing under anaerobic conditions

**Fig. 5:**
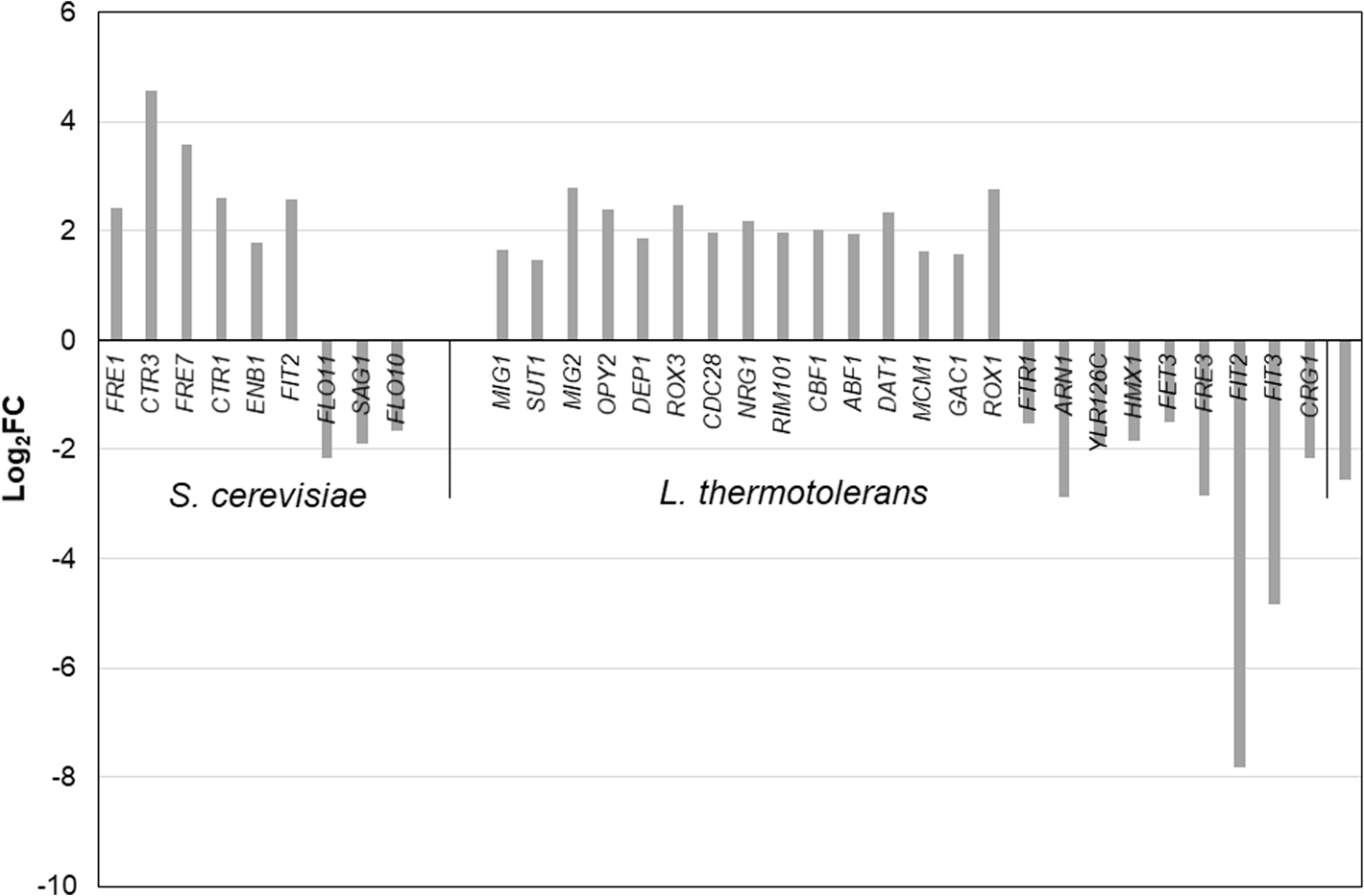
Differentially expressed genes involved in enriched biological processes in *S. cerevisiae* and *L. thermotolerans* in response to mixing under anaerobic conditions compared to aerobic conditions

Since the mixed aerobic fermentation samples contained a low percentage of *L. thermotolerans* reads and transcripts, which reduced the power to detect differentially expressed genes, we only considered the mixed culture fermentations under anaerobic conditions for further analyses. The transcriptional response of the two yeasts to mixing was analysed by comparing the mixed culture transcriptome to the monocultures in these conditions (i.e. Sc-Lt-AN vs Sc-AN and Lt-AN). The data revealed that only 62 genes were differentially expressed (31 up-regulated and 31 down-regulated) in *S. cerevisiae* in response to the presence of *L. thermotolerans*. In contrast, in *L. thermotolerans* 687 genes were differentially expressed, amongst them 639 (408 up-regulated and 231 down-regulated) which could be annotated to *S. cerevisiae* homologs. The DEGs were independently analysed for enriched GO terms at the three hierarchical categories (biological process, molecular function and cellular component). Up-regulated genes in *S. cerevisiae* were mainly associated with copper and iron ion import, as well as iron ion homeostasis, while the down-regulated genes were associated with cell aggregation and flocculation (Table 2). The genes significantly up-regulated in the copper and iron homeostasis were *FRE1* and *FRE7* which encode ferric reductases, *CTR1* and *CTR3* encoding high affinity copper transporters, as well as *ENB1* and *FIT2* that encode an endosomal ferric enterobactin transporter and a mannoprotein, respectively. In contrast, *FLO10*, *FLO11* and *SAG1* required for pseudohyphal and invasive growth, as well as flocculation and agglutination, respectively, were down-regulated (Fig. 5). In *L. thermotolerans*, biological processes associated with filamentous growth in response to starvation were enriched with up-regulated genes, while processes associated with iron assimilation, iron ion homeostasis as well as siderophore transport were enriched with down-regulated genes (Table 3). The data show that transcriptional factors and repressors (e.g. *MIG1*, *MIG2*, *OPY2*) that are likely involved in the regulation of filamentous growth were up-regulated, while *FTR1*, *FRE3*, *FET3* and *ARN1* encoding a high affinity iron permease, ferric reductase, ferro-O_2_-oxidoreductase, as well as ARN family transporter for siderophore-iron chelates, respectively, were down-regulated (Fig. 5).

**Table 2:** Enriched GO terms (Biological Processes (BP), Biological Function (BF) and Cellular component (CC)) in *Saccharomyces cerevisiae* in anaerobic mixed fermentations

Expression	Category	GO Terms	FDR q-value	Description
Up regulated	BP	GO:0015677	1.70E-04	Copper ion import
		GO:0000041	9.06E-04	Transition metal ion transport
		GO:0006825	8.15E-04	Copper ion transport
		GO:0030001	8.85E-03	Metal ion transport
		GO:0006826	2.82E-02	Iron ion transport
		GO:0055072	3.28E-02	Iron ion homeostasis
		GO:0055076	1.26E-01	Transition metal ion homeostasis
		GO:0035434	1.50E-01	Copper ion transmembrane transport
		GO:0055065	2.84E-01	Metal ion homeostasis
		GO:0015688	3.12E-01	Iron chelate transport
		GO:0015891	2.83E-01	Siderophore transport
		GO:0071555	3.78E-01	Cell wall organization
		GO:0045229	3.49E-01	External encapsulating structure organization
	BF	GO:0005199	1.70E-02	Structural constituent of cell wall
		GO:0005375	1.36E-01	Copper ion transmembrane transporter activity
		GO:0052851	1.90E-01	Ferric-chelate reductase (NADPH) activity
		GO:0000293	1.89E-01	Ferric-chelate reductase activity
		GO:0016723	1.51E-01	Oxidoreductase activity, oxidizing metal ions, NAD or NADP as acceptor
		GO:0016811	2.35E-01	Hydrolase activity, acting on carbon-nitrogen (but not peptide) bonds, in linear amides
		GO:0016722	3.47E-01	Oxidoreductase activity, oxidizing metal ions
	CC	GO:0009277	4.84E-02	Fungal-type cell wall
		GO:0030312	3.04E-02	External encapsulating structure
		GO:0005618	2.30E-02	Cell wall
Down-regulated	BP	GO:0098743	8.92E-03	Cell aggregation
		GO:0098630	4.46E-03	Aggregation of unicellular organisms
		GO:0051704	6.15E-03	Multi-organism process
		GO:0098609	8.21E-02	Cell-cell adhesion
		GO:0000128	9.84E-06	Flocculation
	BF	GO.0035673	1.56E-01	Oligopeptide transmembrane transporter activity
		GO:1904680	1.00E+ 00	Peptide transmembrane transporter activity
	CC	GO:0005576	2.42E-04	Extracellular region
		GO:0030312	7.10E-03	External encapsulating structure
		GO:0005618	4.74E-03	Cell wall
		GO:0031225	6.22E-03	Anchored component of membrane
		GO:0000322	1.08E-02	Storage vacuole
		GO:0000324	8.99E-03	Fungal-type vacuole

**Table 3:** Enriched GO terms (Biological Processes (BP), Biological Function (BF) and Cellular component (CC)) in *Lachancea thermotolerans* under anaerobic mixed fermentations

Expression	Category	GO Terms	FDR q-value	Description
Up regulated	BP	GO:0005975	9.65E-03	Carbohydrate metabolic process
		GO:0044281	8.15E-03	Small molecule metabolic process
		GO:0035434	4.67E-02	Copper ion transmembrane transport
		GO:0044282	4.19E02	Small molecule catabolic process
		GO:0043436	4.79E02	Oxoacid metabolic process
		GO:0006082	4.34E-02	Organic acid metabolic process
		GO:1900434	3.91E-02	Regulation of filamentous growth of a population of unicellular organisms in response to starvation
		GO:0019752	3.79E-02	Carboxylic acid metabolic process
	BF	GO:0001078	1.96E-03	Transcriptional repressor activity, RNA polymerase II proximal promoter sequence-specific DNA binding
		GO:0001227	1.47E-03	Transcriptional repressor activity, RNA polymerase II transcription regulatory region sequence-specific DNA binding
		GO:0000982	5.40E-02	Transcription factor activity, RNA polymerase II proximal promoter sequence-specific DNA binding
	CC	GO:0005576	1.39E-03	Extracellular region
		GO:0005618	6.98E-02	Cell wall
Down-regulated	BP	GO:0055072	2.97E-05	Iron ion homeostasis
		GO:0055076	4.61E-04	Transition metal ion homeostasis
		GO:0006879	6.37E-04	Cellular iron ion homeostasis
		GO:0048878	1.03E-03	Chemical homeostasis
		GO:0055065	1.98E-03	Metal ion homeostasis
		GO:0015688	2.89E-03	Iron chelate transport
		GO:0015891	2.48E-03	Siderophore transport
		GO:0046916	5.32E-03	Cellular transition metal ion homeostasis
		GO:0051321	5.84E-03	Meiotic cell cycle
		GO:0042592	7.78E-03	Homeostatic process
		GO:1901678	8.35E-03	Iron coordination entity transport
		GO:0098771	8.65E-03	Inorganic ion homeostasis
		GO:0055080	1.04E-02	Cation homeostasis
		GO:0006826	1.09E-02	Iron ion transport
		GO:0050801	1.97E-02	Ion homeostasis
	BF	GO:0016491	5.40E-01	Oxidoreductase activity
		GO:0016722	4.45E-01	Oxidoreductase activity, oxidizing metal ions
		GO:0050662	6.57E-01	Coenzyme binding
	CC	GO:1990351	2.83E-01	Transporter complex
		GO:0097249	1.66E-01	Mitochondrial respiratory chain supercomplex
		GO:0033573	2.19E-01	High-affinity iron permease complex
		GO:19–05862	1.64E-01	Ferroxidase complex

**Fig. 6:**
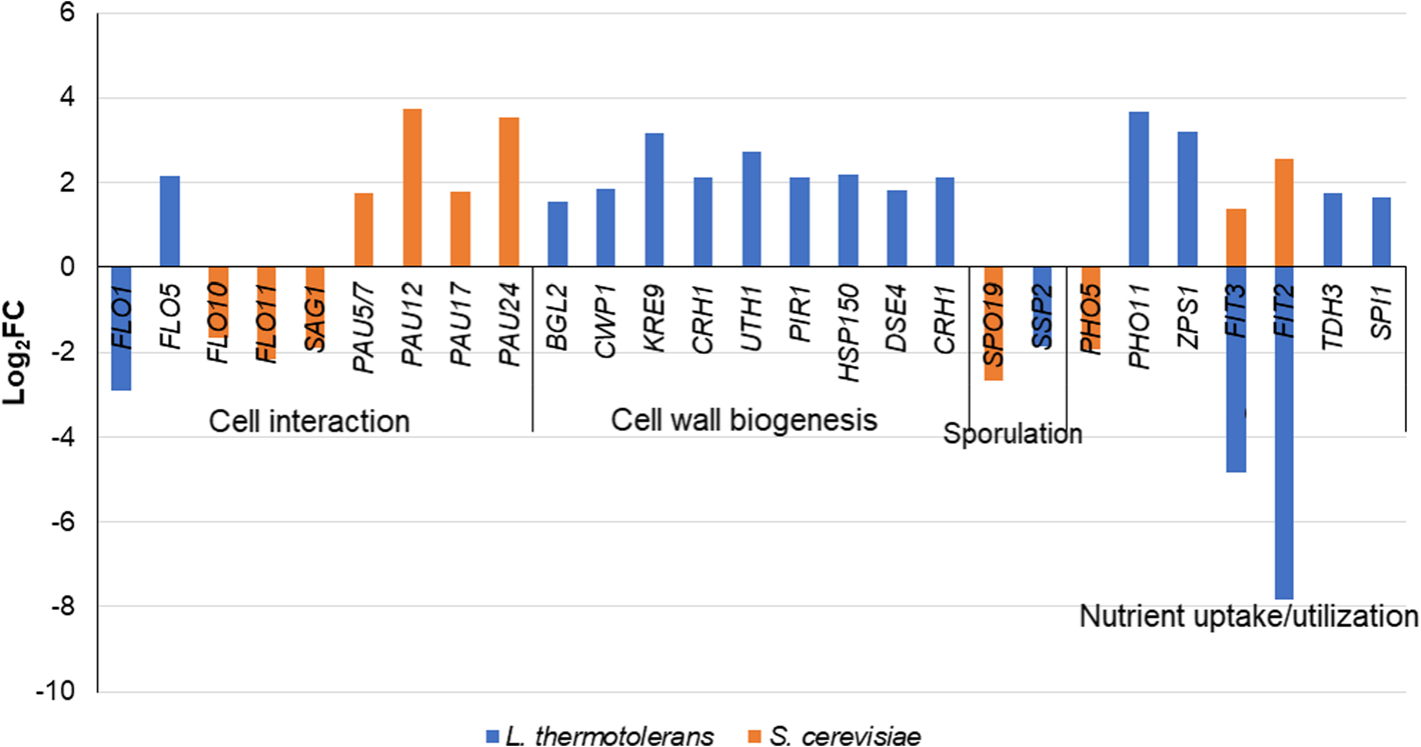
DEGs associated with the enrichment of the cellular component GO terms: cell wall and cell exterior

**Fig. 7:**
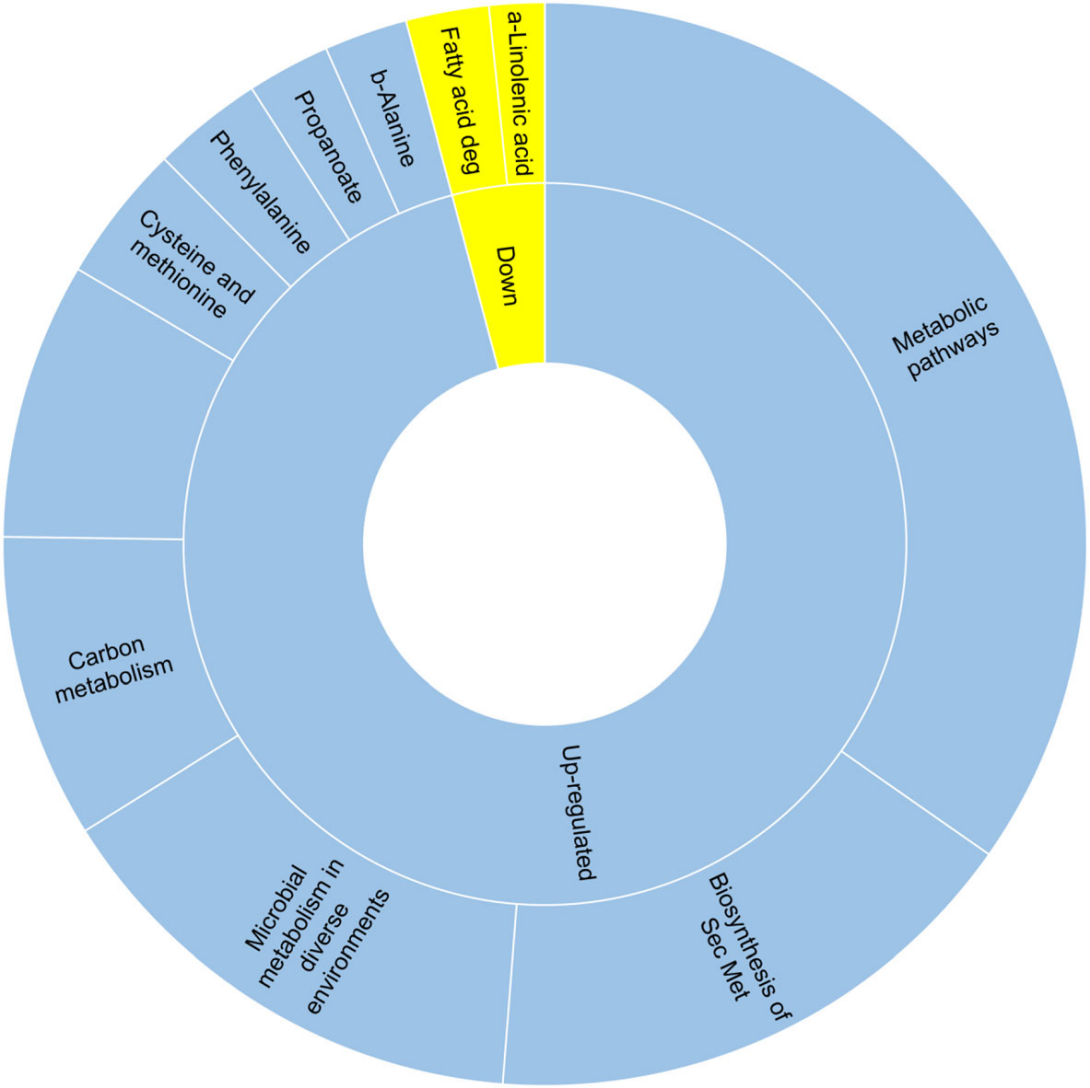
KEGG pathways enriched in *L. thermotolerans* in mixed culture fermentation under anaerobic conditions compared to aerobic conditions

Within the category of Cellular component, GO enrichment revealed that the extracellular region and the cell wall were the most enriched cellular component GO terms in *S. cerevisiae* with both up- and down-regulated genes (Table 2). The enrichment is mainly associated with the up-regulation of mannoprotein encoding genes (*FIT2* and *FIT3*) as well as the seripauperin protein encoding genes (*PAU5*, *PAU12*, *PAU17* and *PAU24*) and down-regulation of *FLO10*, *FLO11*, *SPO19*, *SAG1* and *PHO5* (Fig. 7). Conversely, in *L. thermotolerans* the cell wall was only enriched with up-regulated genes. The genes associated with this enrichment included *KRE9*, *BGL2*, *DSE4*, *CWP1*, *PIR1*, *HSP150* (a paralog of *PIR3*), *UTH1*, and *CRH1* (Fig. 6) all required for cell wall biogenesis and stability, particularly β-glucan formation.

The DEGs were further mapped to terms in the Kyoto Encyclopedia of Genes and Genomes (KEGG, http://www.kegg.jp/) database. No pathways were enriched in *S. cerevisiae* probably due to the limited number of genes (62) differentially expressed in response to mixing. Conversely, in *L. thermotolerans* out of the 639 annotated DEGs,116 up-regulated and 5 down-regulated genes revealed enriched KEGG pathways. As illustrated in Fig. 7, metabolic pathways, biosynthesis of secondary metabolites as well as microbial metabolism in diverse environments were the largest categories in the up-regulated genes. Furthermore, the data show that carbon metabolism as well as biosynthesis of amino acids were highly enriched. Moreover, the metabolism of β-alanine, phenylalanine, cysteine and methionine was enriched. However, not all the genes involved in the metabolic processes were significantly upregulated. For instance, in the β-alanine metabolism only *PAN6* and *GAD1* were significantly up-regulated, while *ARO8* and *AAT2*, and *CYS3*, *ARO8*, *MCY1* and *AAT2*, were significantly up-regulated in the phenylalanine and the cysteine and methionine metabolism, respectively (Additional file 4: Table S4). Amongst the down-regulated genes, fatty acid degradation as well as α-linoleic acid metabolism were the main enriched pathways.

## Discussion

The current study aimed to unravel transcriptional responses of *S. cerevisiae* and *L. thermotolerans* in mixed culture fermentation. By manipulating dilution rates in a continuous culture, the two yeasts were maintained at approximately equal concentrations for 24 h. *L. thermotolerans* transcripts accounted for 24% of the sequences reads in anaerobic mixed cultures and ≈ 8% in aerobic mixed cultures. However, there was sufficient depth in the anaerobic mixed fermentation transcriptome to extract key genes that are influenced by mixing and interaction between *S. cerevisiae* and *L. thermotolerans*. Related studies have employed strategies, which only allowed for analysis of early transcriptional responses to mixed culture or analyses of *S. cerevisiae* transcriptional response only. For instance, Tronchoni et al. [25] first pre-cultured *S. cerevisiae* and the *T. delbrueckii* in separate bioreactors and then mixed the two at equal volumes, followed by withdrawal of samples for RNAseq before the cells started proliferating and in the early exponential phase to avoid over-representation of only one species. In contrast, Barbosa et al. [17] conducted mixed-culture fermentations of *S. cerevisiae* with *Hanseniaspora guilliermondii* in a typical wine fermentation batch set-up, extracted RNA at different fermentation stages and only focused on the transcriptional response of *S. cerevisiae*. The specific conditions also aimed at maintaining relatively stable environmental conditions and cell densities during the co-culture of the two species by using a set-up similar to a continuous fermentation. This strategy was intended to limit the transcriptomic signature to the interaction-relevant responses by reducing genetic responses that would be due to the continuous dynamic adaptation to changing conditions that are prevalent in batch cultures [17, 25]. The relatively limited number of *S. cerevisiae* genes that are significantly affected by the mixed culture conditions in our study when compared to other such data sets suggests that this strategy has succeeded, and that the highlighted genes are indeed the core of a perpetual interaction response.

### Overall response to the interaction between mixing and anoxia

The transcriptomic data revealed divergent responses of the two yeasts to the interaction between mixing and anoxia. These conditions elicited a stronger response in *L. thermotolerans*. Indeed, on average *L. thermotolerans* displayed a marked up-regulation of genes associated with cell aggregation, cell death and response to osmotic stress. Furthermore, several genes encoding catalase, superoxide dismutases, and peroxidases were significantly up-regulated, suggesting that *L. thermotolerans* cells are experiencing some level of oxidative stress. This is not surprising since other studies have demonstrated that hypoxia and anoxia induce transient oxidative stress in other yeasts such as *S. cerevisiae* and *Kluyveromyces lactis* [26]. Our data also suggest that *L. thermotolerans* redirects its metabolic flux from glycolysis to the PPP similar to *K. lactis* and *Pichia pastoris* [26, 27].

Under anoxic conditions, mitochondrial and cytochrome c activities were down-regulated in both yeasts. However, differences were observed in the up-regulated processes, where seemingly *S. cerevisiae* increased its sterol uptake while *L. thermotolerans* increased the expression of genes required for ergosterol biosynthesis including those encoding enzymes that catalyse oxygen-dependent reactions e.g. *ERG3*, *ERG11* and *ERG25*. This induction of oxygen-dependent genes has been observed in other yeasts including *S. cerevisiae* and *P. pastoris* albeit transiently, and has been attributed to cells adjusting to anaerobic conditions following cultivations in oxygenated environments [27].

### Transcriptional responses to mixed culture fermentation – Iron and copper acquisition

In the current study, it was evident that in mixed-culture fermentation, *S. cerevisiae* increased the expression of iron and copper acquisition systems. Indeed, the data show that *FRE1*, *FRE7*, *ENB1*, *FIT2*, *CTR1* and *CTR3* were significantly upregulated. Fre1p, Enb1p and Fit2p form a cluster of proteins required for the uptake of siderophores. Fre1p has broad substrate specificity and can catalyse the reduction of iron-sidephore chelates such as ferrichrome, ferrioxamine B and enterobactin. Enb1p exclusively recognizes and transports enterobactin, while Fit2p contributes to the retention of siderophore-iron chelates in the cell wall [28]. Regarding copper uptake, Ctr1p and Ctr3p together with Fre1p and Fre7p are important in high affinity uptake of copper ions. Their expression is a cellular response to inadequate intracellular copper levels [29]. Conversely, *L. thermotolerans* shown a down-regulation of genes encoding nonreductive (copper independent) siderophore-iron transport (*ARN1*, *FIT2*, *FIT3*), as well as those required for reductive iron uptake (FRE3) and copper-dependent iron import (*FTR1* and *FET3*). Fet3p is multicopper oxidase that also exhibit ferrous oxidase activity and forms a high-affinity iron transport complex with Ftr1p as well as Fet5p and Fth1p [30], which were not differentially expressed in the current study. Hodgins-Davis et al. [31] showed that *FTR1* and *FET3* were uniformly downregulated under copper deprivation in different *S. cerevisiae* strains. Since high-affinity copper and iron acquisition systems are homeostatically regulated, we can infer from the current data that both yeasts experience the growth conditions to be limited in bioavailable iron and copper, and therefore activate different systems to allow them to thrive in such environments. In *S. cerevisiae,* high expression of *CTR1* gene is reported to be induced when copper levels are below 10 μM [30], while high concentrations of copper result in down-regulation of both *CTR1* and *CTR3* [29]. The synthetic grape juice medium used in the current study contains 0.11 μM copper. Copper is required for iron homeostasis in yeast and the link between copper and iron metabolism in *S. cerevisiae* is well recognized [29]. Evidently, in mixed-culture fermentation, *S. cerevisiae* competes with *L. thermotolerans* by activating a full set of genes to acquire different forms of iron from the environment and to store it in the cells in bound-form (e.g. ferrichrome).

### Transcriptional responses to mixed culture fermentation – Cell wall integrity and adhesion

Our data show a strong response to cell wall integrity in both yeasts in mixed fermentation when compared to single species fermentation. Indeed, both yeast species up-regulate genes that are involved in cell wall integrity under stress conditions although different genes and processes are induced. Indeed, under anaerobic mechanisms in mixed fermentations. In particular, *S. cerevisiae* shows a significant up-regulation of 5 *PAU* genes (*PAU5, PAU9, PAU12, PAU17, PAU24*). These genes have been reported to play an important role in promoting fitness under anaerobic and fermentative condition as well as in interactions between natural strains of *S. cerevisiae* [32, 33]. In particular, *PAU5* was shown to play a key role in competition by providing protection against killer toxins [33]. The abundance of the PAU genes in the *S. cerevisiae* transcriptome in the presence of *L. thermotolerans* could therefore suggest that they also play a role in interactions with other yeast species which are phylogenetically closely related to *S. cerevisiae*. Indeed, in another study, Tronchoni et al. [25] reported an induction of 20 of the 24 *PAU* genes in the primary response of *S. cerevisiae* to *Torulaspora delbrueckii*. Our data suggest that these genes are indeed specifically associated with yeast-yeast interactions. Interestingly, in the mixed fermentation under anaerobic conditions, *L. thermotolerans* increases the expression of endoglucanases (e.g. *BGL2*), while simultaneously reinforcing and stabilizing its cell wall, as evident in the up-regulation of genes involved in the biogenesis, assembly and maintenance of glucan and chitin. The overexpression of *BGL2* in *S. cerevisiae* has been shown to retard growth, which could suggest that *L. thermotolerans* compensates for this possible impact by strengthening the cell wall. In contrast, *S. cerevisiae* protects itself through the upregulation of *PAU* genes, which are amongst the genes that encode cell wall proteins thought to be important in cell wall remodelling and maintenance of cell wall integrity during stress [32]. In the current study, the recovery of *L. thermotolerans* reads in mixed culture fermentations was consitently lower than those of *S. cerevisiae* (Additional file 1: Table S1). We may deduce from our data that this alludes to poor RNA extraction which could partly be due to cell wall thickness and rigidity, that rendered the *L. thermotolerans* cells less sensitive to mechanical disruption. However, this will require further evaluation.

### Transcriptional responses to mixed culture fermentation – Amino acid metabolism

Our data suggest that in mixed culture fermentation under anaerobic conditions, *L. thermotolerans* specifically increases the expression of genes involved in the metabolism of four amino acids viz. cysteine, methionine, phenylalanine and β-alanine. The up-regulation of cystein and methionine-related metabolic activities may suggest that sulphur-related processes are directly affected by co-culture conditions. Furthermore, all genes involved in the conversion of phenylalanine to phenylethanol were up-regulated. These data support our previous findings which showed that *L. thermotolerans* produces high levels of phenylethanol in monoculture, and to enhance phenylethanol production in mixed fermentations with *S. cerevisiae* [5]. This trait seems common in various *L. thermotolerans* since other studies have reported increased levels of phenylethanol in combinations of different strains of *L. thermotolerans* and *S. cerevisiae* [10, 11].

## Conclusions

Overall, our study reveals divergent molecular signatures underlying the performance of *S. cerevisiae* and *L. thermotolerans* in mixed culture fermentation. The data shows that *S. cerevisiae* is better able to deal with the fermentation environment possibly due to its efficient competitive uptake of sterols, copper and iron, accompanied by cell wall remodelling to accommodate additional mannoproteins and PAU proteins. These strategies allow the yeast to regulate membrane fluidity and cell wall porosity, and withstand an anaerobic, high ethanol environment. Conversely, the fermentation environment seems highly toxic to *L. thermotolerans*, which mainly features a molecular signature that is characterized by detoxification, cell aggregation and cell death associated genes. The strong cell wall-related responses in both species suggest the importance of this organelle in the cellular response to other species. In particular, the data support that the regulation of adhesion properties may play a central role in modulating the physical and ecological interactions between species [16].

The data are also a confirmation of many studies that have reported a rapid decline of *L. thermotolerans* in wine fermentation especially in mixed cultures with *S. cerevisiae*. The study also underlines the usefulness of a global approach to the study of yeast-yeast interactions to shed light on the molecular basis of yeast dynamics during wine fermentation. Besides a general contribution to a better understanding of yeast ecological interactions, the data will be useful for the rational development of mixed-starter cultures in the winemaking industry.

## Methods

### Yeast strains and media

*S. cerevisiae* (Cross evolution-285) was obtained from Lallemand SAS (Blagnac, France), while *L. thermotolerans* (IWBT-Y1240) was obtained from the culture collection of the Institute for Wine Biotechnology (Stellenbosch University). Yeast strains were maintained cryogenically (− 80 °C) and were reactivated by streaking out on YPD agar plates containing (per litre) 10 g yeast extract, 20 g peptone and 20 g glucose and 20 g bacteriological agar. Cultures were stored at 4 °C for short-term use.

### Batch fermentation

Batch fermentations were performed in synthetic grape juice medium containing (per litre) 100 g glucose, 100 g fructose (Merck), 1 g yeast extract (Oxoid), 0.3 g citric acid, 5 g l-malic acid, 5 g l-tartaric acid, 0.4 g MgSO_4_, 5 g KH_2_PO_4_, 0.2 g NaCl, 0.05 g MnSO_4_ (Sigma-Aldrich) and anaerobic factors (ergosterol 10 mg (Sigma-Aldrich), tween 80 0.5 mL (Merck)) [34, 35]. Fermentations were conducted in 1.3 L BioFlo 110 bench top bioreactors (New Brunswick, NJ, USA) using 900 mL of final working volume, a temperature of 25 °C and an agitation speed of 200 rpm. Fermentations were performed under two conditions: anaerobic and aerobic at 5% (0.41 mg L^− 1^) dissolved oxygen (DO). The anaerobic conditions were created by initially sparging N_2_ to bring down the DO level to 0%, and then to minimize diffusion of atmospheric oxygen into the cultures, the entire fermentation set-up was equipped with Norprene tubing. The 5% DO level was maintained through the supplementary addition of 4 gasses (CO_2_, N_2_, O_2_ and compressed air) whenever required, using an automated gas flow controller. The DO levels in the cultures were monitored with an oxygen electrode.

### Fermentation conditions

In order to maintain similar environmental conditions in mixed and single-culture fermentations, a system similar to continuous fermentation using continuous in-flow and out-flow of the medium was optimised for single and mixed fermentations. Samples for RNAseq analysis were withdrawn at 48 h when total viable cell count was similar between the mixed and single culture fermentation. The feeding medium contained glucose and fructose, each at 50 g L^− 1^. The working volume was maintained at 0.7 L using a peristaltic effluent pump. All fermentations were conducted in duplicate.

### Analysis of population dynamics

Serial dilutions of the cell suspensions were performed with 0.9% (*w*/*v*) NaCl. One hundred microliter samples were spread on YPD agar and incubated at 30 °C for 2–3 days. For yeast enumeration in mixed culture fermentations, both species were distinguished based on colony morphology. Colony counts were performed on plates with 30–300 colonies.

### Analytical methods

Supernatants were obtained by centrifuging cell suspensions at 5000×*g* for 5 min. The concentrations of fructose, glucose, acetaldehyde and acetic acid were measured using specific enzymatic kits, Enytec™ *Fluid* d-fructose, glucose, acetic acid (Thermo Fisher Scientific Oy, Finland), Boehringer Mannheim / R-Biopharm-acetaldehyde (R-Biopharm AG, Darmstadt) and analyzed using Arena 20XT photometric analyzer (Thermo Electron Oy, Helsinki, Finland). Ethanol was analysed by high performance liquid chromatography (HPLC) on an AMINEX HPX-87H ion exchange column using 5 mM H_2_SO_4_ (Sigma-Aldrich) as the mobile phase as described by Rossouw et al. [24]. The major volatiles were extracted with diethyl ether and analysed by Gas Chromatography with Flame Ionization Detection (GC-FID) as described in previously [36].

### Sampling, RNA-extraction and RNA-sequencing

Cell samples for RNA-sequencing were obtained from both single and mixed culture fermentations (anaerobic and aerobic, respectively) at 48 h when population and sugar levels were approximately same in all fermentations. Total RNA extractions were performed according to the hot phenol method [37]. Concentration and purity of RNA were determined by spectrophotometry and integrity was confirmed using an Agilent 2100 Bioanalyzer with an RNA 6000 Nano Assay (Agilent Technologies, Palo Alto, CA, USA). The RNA samples with RNA integrity number (RIN) more than 8, and 280:260 ratios more than 2 were further used for the RNA-sequencing purpose. Library preparation and sequencing was performed by VIB Nucleomics core (KU, Leuven (Belgium). Complementary DNA (cDNA) library was generated using TruSeq® Library Prep Kit v2. Paired-end reads were sequenced on the Illumina NextSeq platform.

### RNAseq data processing

Low quality reads (< Q20), polyA-reads as well as ambiguous reads (containing N) were removed using FastX 0.0.13 [38]. Furthermore, reads shorter than 35 bp were removed with ShortRead 1.16.3 [39] and adapters on the remaining reads were trimmed with cutadapt 1.7.1 [40].

### RNAseq data analysis

Annotation of genomic features was performed using the reference genomes of *S. cerevisiae* S288c and *L. thermotolerans* CBS6340. In the case of *L. thermotolerans* unknown genes were identified by the homology with the *S. cerevisiae* S288c genome. Reads from *L. thermotolerans* and *S. cerevisiae* monoculture fermentation samples were aligned to the reference genomes of the two yeasts with TopHat v2.0.13 [41]; and reads that were non-primary mapping or had a mapping quality ≤20, were removed. Subsequently, cross-mapping between *S. cerevisiae* S288c and *L. thermotolerans* was evaluated to determine the impact of merging the genomes. Cross-mapping between the two yeasts was found to be less than 1%; consequently, pre-processed reads of all fermentations were aligned to the reference genome of S288cplusLT. The obtained *bam* files were further converted in to *gff* files to analyse the data further. The number of reads in the alignments that overlap with gene features were counted using htseq-count 0.6.1p1 [42]. Genes for which all samples had less than 1 count-per-million were removed and full quantile normalization using the *EDASeq* package from Bioconductor was applied to correct for sample-specific variation typically introduced by differences in library size and RNA composition. Transcript abundance was measured in Fragments Per Kilobase of exon per Million mapped reads (FPKM).

### Identification and statistical analysis of differentially expressed genes

For the selection of differentially expressed genes statistical modelling was used to design the following experiments:

$$ \log (Count)=\beta 1+ MIX\times \beta 2+ AN\times \beta 3+ INT\times \beta 4 $$

For each gene the coefficients β were estimated with the edgeR 3.8.6 package of Bioconductor [43], by fitting a negative binomial generalized linear model (GLM) [44]. Offsets were used to estimate the models. Subsequently, the model estimates were used to compute contrasts of primary interest which were (i) MIX vs PURE (MIX effect) (ii) AN vs AR (AN effect), (iii) the interaction between MIX and AN effect (INT), (iv) MIX vs PURE, only in AR samples, (v) MIX vs PURE, only in AN samples and (vi) AN vs AR, only in MIX samples. The differential expression was tested with a GLM likelihood ratio test, also implemented in the edgeR 3.8.6 package. The resulting *p*-values were corrected for multiple testing with Benjamini-Hochberg to control the false discovery rate (FDR) [45]. Genes with an absolute log2-ratio larger than 1 and an adjusted *p*-value < 0.05 were considered differentially expressed.

## Additional files

Additional file 1: **Table S1.** The table provides details of number of reads per sample sequenced and after number of reads used to analyse the data after removing bad sequences and reads less than 35 bp (DOCX 27 kb)

Additional file 2: **Table S2.** A list of genes differentially expressed in *Saccharomyces cerevisiae* and *Lachancea thermotolerans* in mixed fermentations compared to monocultures. (XLSX 1539 kb)

Additional file 3: **Table S3.** A summary of genes differentially expressed in different metabolic processes in *Saccharomyces cerevisiae* and *Lachancea thermotolerans* due to mixing and the interaction between mixing and anoxia (DOCX 49 kb)

Additional file 4: **Table S4.** Up-regulated genes involved in the metabolism of cysteine, methionine, phenylalanine and β-alanine in *Lachancea thermotolerans (DOCX 28 kb)*

## Abbreviations

ADP: Adenosine di-phosphate
AN: Anaerobic
AR: Aerobic
ATP: Adenosine tri-phosphate
BF: Biological function
BP: Biological processes
CC: Cellular component
cDNA: Complementary DNA
CFU: Colony forming units
DEG: Differentially expressed genes
DNA: Deoxyribonucleic acid
DO: Dissolved oxygen
FC: Fold change
FDR: False discovery rate
FPKM: Fragments per kilobase of exon per million mapped reads
GC-FID: Gas chromatography with flame ionization detection
GEO: Gene expression omnibus
GLM: Generalized linear model
GO: Gene ontology
HPLC: High performance liquid chromatography
IWBT: Institute for wine biotechnology
KEGG: Kyoto encyclopedia of genes and genomes
Lt: *Lachancea thermotolerans*
Mb: Million base
NADP: Nicotinamide adenine dinucleotide phosphate
NCBI: National Centre for Biotechnology Information
NRF: National Research Foundation
PCA: Principal component analysis
RIN: RNA integrity number
RNA: Ribonucleic acid
Sc: *Saccharomyces cerevisiae*
TPM: Transcripts per million
YPD: Yeast potato dextrose
