# Supplementary material for: RNA-seq based transcriptional analysis of Saccharomyces cerevisiae and Lachancea thermotolerans in mixed-culture fermentations under anaerobic conditions
Source: BMC Genomics. 2019 Feb 18;20:145. doi: 10.1186/s12864-019-5511-x (PMC6379982; doi:10.1186/s12864-019-5511-x)
Supplement: Supplementary file 1 — Table S1. The table provides details of number of reads per sample sequenced and after number of reads used to analyse the data after removing bad sequences and reads less than 35 bp (DOCX 27 kb) [file 12864_2019_5511_MOESM1_ESM.docx]

Additional file

**Table S1** The table provides details of number of reads per sample sequenced and after number of reads used to analyse the data after removing bad sequences and reads less than 35bp

| **Sample name** | **Total number of initial paired reads**  **(Mb)** | **Total number of paired reads after pre-processing**  **(Mb)** | **Total number of remaining Sc reads**  **(Mb)** | **Total number of remaining Lt reads**  **(Mb)** |
| --- | --- | --- | --- | --- |
| Lt-AN-1 | 13,103,758 | 12,894,262 |  | 10,635,170 |
| Lt-AN-2 | 12,690,664 | 12,489,747 |  | 10,416,845 |
| Lt-AR-1 | 13,188,156 | 12,988,306 |  | 10,800,720 |
| Lt-AR-2 | 13,259,623 | 13,038,647 |  | 11,0581,62 |
| Sc-AN-1 | 12,184,573 | 11,270,192 | 9,186,170 |  |
| Sc-AR-1 | 13,441,701 | 13,210,436 | 6,647,398 |  |
| Sc-AR-2 | 14,643,317 | 14,398,120 | 12,297,256 |  |
| Sc+Lt-AN-1 | 15,131,860 | 14,831,934 | 9,405,822 | 2,943,362 |
| Sc+Lt-AN-2 | 14,101,375 | 13,839,143 | 8,797,326 | 2,788,971 |
| Sc+Lt-AR-1 | 15,421,107 | 15,177,399 | 11,998,758 | 941,753 |
| Sc+Lt-AR-2 | 14,917,699 | 14,617,544 | 11,2473,81 | 1,064,516 |
