# Supplementary material for: RNA-seq based transcriptional analysis of Saccharomyces cerevisiae and Lachancea thermotolerans in mixed-culture fermentations under anaerobic conditions
Source: BMC Genomics. 2019 Feb 18;20:145. doi: 10.1186/s12864-019-5511-x (PMC6379982; doi:10.1186/s12864-019-5511-x)
Supplement: Supplementary file 3 — Table S3. A summary of genes differentially expressed in different metabolic processes in Saccharomyces cerevisiae and Lachancea thermotolerans due to mixing and the interaction between mixing and anoxia (DOCX 49 kb) [file 12864_2019_5511_MOESM3_ESM.docx]

**Table S3: A summary of genes differentially expressed in different metabolic processes in *Saccharomyces cerevisiae* (Sc) and *Lachancea thermotolerans* (Lt) due to mixing and the interaction between mixing and anoxia**

| **Category** | **Gene Name** | **Log_2_FC** | | | |
| --- | --- | --- | --- | --- | --- |
|  |  | Sc-mix | Sc-Int | Lt-mix | Lt-int |
| Pentose Phosphate Pathway | *SOL3* | -0.2 | -0.2 | -0.1 | 3.5 |
|  | *SOL4* | -0.2 | -0.4 |  |  |
|  | *SOL2* | -0.6 | 1.4 |  |  |
|  | *SOL1* | 0.1 | -0.6 | -0.2 | 0.6 |
|  | *ZWF1* | -0.1 | 2.3 | 0.01 | -0.6 |
|  | *GND1* | -0.03 | 1.2 | -0.01 | 2.1 |
|  | *TAL1* | -0.9 | 1.7 |  |  |
|  | *NQM1* | 0.2 | 0.5 |  |  |
|  | *TKL2* | 0.02 | 0.7 | -0.2 | 1.4 |
|  | *TKL1* | -0.3 | 0.3 | -0.2 | 3.3 |
|  | *RPE1* | -0.4 | -2.1 | -0.2 | 0.6 |
| Secondary Metabolites Degradation | *SOR1* |  |  | 0.28 | 2.5 |
|  | *HXK1* | -0.2 | 4.0 |  |  |
|  | *HXK2* | 0.1 | 1.0 | 0.1 | 0.2 |
|  | *XYL2* | 0.9 | 1.4 |  |  |
|  | *XKS1* | 0.4 | 1.4 | 0.2 | 0.5 |
| Alcohols Degradation | *BDH1* | -0.8 | 2.6 | 0.5 | -0.5 |
|  | *ADH2* | -0.6 | 1.8 |  |  |
|  | *ALD5* | -1.1 | 0.6 | -0.1 | 3.2 |
|  | *ALD4* | 0.8 | -0.9 | 0.6 | 0.7 |
|  | *ACS1* | 0.4 | -0.3 |  |  |
|  | *ACS2* | 0.3 | 0.9 | 0.4 | 1.4 |
|  | *GUT1* | -0.1 | -0.6 | -0.4 | 3.9 |
|  | *PGC1* | 0.6 | 0.4 | 0.2 | -0.9 |
|  | *GDE1* | 0.2 | 0.2 | 0.5 | 1.7 |
|  | *GUT2* | 0.6 | -0.5 | -0.2 | -0.5 |
| Amines and Polyamines Biosynthesis | *PCT1* | -0.2 | 0.5 | -0.2 | 4.8 |
|  | *SPO14* | 0.3 | 0.7 | -0.1 | 1.7 |
|  | *EPT1* | 0.1 | 0.6 |  |  |
|  | *CPT1* | 0.4 | -0.3 | 0.2 | -0.7 |
|  | *SPE1* | -0.1 | -0.7 | -0.02 | 0.7 |
|  | *SPE3* | 0.2 | -1.6 | -0.1 | -0.01 |
|  | *SPE2* | -0.1 | -0.3 | -0.4 | 1.0 |
|  | *SPE4* | -0.1 | -1.0 | -0.2 | -0.2 |
| Metabolic Regulators Biosynthesis | *FBP26* | 0.2 | 0.7 |  |  |
|  | *YLR345W* | 0.2 | 1.4 |  |  |
|  | *PFK26* | 0.2 | 0.9 |  |  |
|  | *PFK27* | 0.03 | 0.4 |  |  |
|  | *TSL1* | -0.1 | 3.7 |  |  |
|  | *TPS1* | -0.04 | 2.2 |  |  |
|  | *TPS2* | -0.2 | 2.2 |  |  |
|  | *TPS3* | 0.2 | 1.4 |  |  |
| Detoxification | *CYB2* | 0.1 | -2.3 | 0.6 | -2.3 |
|  | *GRE2* | 0.2 | -0.5 | -0.9 | 1.3 |
|  | *ALD2* | 1.2 | -0.4 | 1.9 | 1.9 |
|  | *ALD3* | 0.9 | 0.03 |  |  |
|  | *ALD5* | -1.1 | 0.6 | -0.1 | 3.2 |
|  | *ALD4* | 0.8 | -0.9 | 0.6 | 0.7 |
|  | *GLO1* | 0.2 | 1.2 | -0.1 | -0.2 |
|  | *GLO4* | -0.01 | -0.2 | -0.01 | 0.5 |
|  | *GLO2* | -0.004 | 0.6 |  |  |
|  | *DLD2* | 0.1 | -0.9 | -0.03 | 0.7 |
|  | *DLD1* | 0.2 | 1.2 | -1.6 | 5.2 |
|  | *DLD3* | -0.5 | 1.2 |  |  |
|  | *SOD1* | -1.0 | -0.2 | -1.7 | 3.2 |
|  | *SOD2* | 0.1 | 1.2 | -0.6 | 2.7 |
|  | *CTA1* | -0.7 | -0.5 | -0.7 | 0.3 |
|  | *CTT1* | -0.3 | -0.6 | -0.2 | 2.3 |
| Proteins involved in Cold response | *CDC28* | -0.004 | 0.2 | -1.1 | 6.1 |
|  | *DBP5* | -0.1 | -0.8 | -0.1 | 0.1 |
|  | *MGA2* | -0.1 | 0.9 | -0.1 | 0.9 |
|  | *SPT23* | -0.1 | -0.02 |  |  |
|  | *IRA2* | -0.1 | 0.4 |  |  |
|  | *ATH1* | 0.5 | 0.1 | 0.4 | 0.7 |
|  | *TIR2* | -0.8 | 3.9 |  |  |
|  | *MSN4* | 0.3 | 0.7 | -0.2 | 0.1 |
|  | *MSN2* | -0.01 | 0.6 |  |  |
| Proteins involved in Cell Death | *YMR074C* | 0.1 | -0.2 | -1.6 | 6.4 |
|  | *MCD1* | 0.4 | 0.5 | -0.05 | -0.1 |
|  | *TIM18* | -0.03 | -0.6 | -0.2 | -1.0 |
|  | *MCA1* | -0.5 | 1.1 | 0.03 | 1.4 |
|  | *LOT6* | 0.2 | 0.2 | 0.2 | -1.8 |
|  | *YSP2* | -0.2 | -0.2 | -0.1 | 0.1 |
|  | *OYE2* | 0.05 | 0.2 | -3.1 | 0.3 |
|  | *ESP1* | 0.1 | 0.1 | 0.03 | -0.6 |
|  | *RNY1* | 0.1 | 1.0 | 1.0 | -1.3 |
|  | *NMA111* | 0.03 | -0.4 | -0.02 | -0.4 |
|  | *AIM14* | 0.5 | -0.4 | -0.5 | -0.8 |
|  | *AIF1* | 0.5 | -1.2 | 0.12 | -0.6 |
|  | *BXI1* | -0.2 | 1.3 | -0.3 | 3.1 |
|  | *BIR1* | 0.1 | -0.02 | 0.1 | -0.3 |
|  | *WWM1* | -0.2 | 0.9 | -0.1 | 1.3 |
|  | *OYE3* | -0.1 | -1.0 |  |  |
|  | *FIS1* | 0.1 | 0.4 | -0.4 | 0.4 |
|  | *DRE2* | -0.2 | -0.5 | -0.1 | 1.8 |
|  | *CPR3* | 0.1 | 0.6 | -0.3 | 4.4 |
|  | *PET9* | 1.0 | -0.7 | 0.1 | 0.7 |
|  | *KEX1* | 0.1 | -0.1 | 0.3 | 1.1 |
|  | *POR1* | 0.4 | 2.3 | 0.004 | 4.9 |
|  | *TDH3* | -0.1 | 0.6 | 0.6 | 3.2 |
|  | *TDH2* | -0.4 | 0.7 |  |  |
|  | *HSP60* | 0.6 | 1.2 | -0.2 | 1.8 |
|  | *YBL055C* | -0.4 | 0.02 | -0.7 | 1.9 |
|  | *NUC1* | 0.3 | -0.8 | -0.2 | -0.8 |
| Proteins involved in adhesion | *GAL83* | 0.1 | 0.5 | -0.1 | 1.2 |
|  | *FIG2* | -0.1 | -0.5 | -0.05 | 0.1 |
|  | *HSP12* | -0.8 | -0.8 | -0.1 | 2.8 |
|  | *SNF1* | -0.1 | 0.2 | 0.01 | 0.3 |
|  | *FLO9* | 1.4 | -1.8 | -0.1 | 1.2 |
|  | *FLO5* | 0.8 | -1.6 | 0.6 | 0.4 |
|  | *NRG1* | 0.1 | -0.4 | -0.4 | 5.1 |
|  | *SDS3* | -0.2 | -0.4 | -0.5 | 1.1 |
|  | *NRG2* | 0.1 | 1.7 |  |  |
|  | *FLO11* | -1.8 | -0.6 |  |  |
|  | *CCW12* | -0.02 | -0.05 | -0.2 | 2.1 |
|  | *AGA2* |  |  | -0.3 | -2.0 |
|  | *AGA1* | 0.1 | -1.7 |  |  |
|  | *SAG1* | -1.1 | -1.5 | 0.1 | -0.4 |
