# Supplementary material for: RNA-seq based transcriptional analysis of Saccharomyces cerevisiae and Lachancea thermotolerans in mixed-culture fermentations under anaerobic conditions
Source: BMC Genomics. 2019 Feb 18;20:145. doi: 10.1186/s12864-019-5511-x (PMC6379982; doi:10.1186/s12864-019-5511-x)
Supplement: Supplementary file 4 — Table S4. Up-regulated genes involved in the metabolism of cysteine, methionine, phenylalanine and β-alanine in Lachancea thermotolerans (DOCX 28 kb) [file 12864_2019_5511_MOESM4_ESM.docx]

**Table S4. Up-regulated genes involved in the metabolism of cysteine, methionine, phenylalanine and β-alanine in *Lachancea thermotolerans***

| **KEGG Pathway** | **Protein Homology Sc** | **Gene name** | **Log_2_FC** |
| --- | --- | --- | --- |
| Cysteine and Methionine metabolism | YAL012W | *CYS3* | 1.83 |
|  | YDL078C | *MDH3* | 2.81 |
|  | YER091C | *MET6* | 1.01 |
|  | YGL184C | *STR3* | 1.07 |
|  | YGL202W | *ARO8* | 2.90 |
|  | YGR012W | *MCY1* | 1.92 |
|  | YLR027C | *AAT2* | 1.61 |
|  | YPR118W | *MRI1* | 1.32 |
| Phenylalanine metabolism | YDR242W | *AMD2* | 1.48 |
|  | YGL202W | *ARO8* | 2.90 |
|  | YLR027C | *AAT2* | 1.61 |
|  | YMR170C | *ALD2* | 1.21 |
|  | YLR134W | *PDC5* | 1.34 |
|  | YDL168W | *SFA1* | 1.19 |
| β-Alanine metabolism | YER073W | *ALD5* | 1.44 |
|  | YIL145C | *PAN6* | 1.51 |
|  | YMR170C | *ALD2* | 1.21 |
|  | YMR250W | *GAD1* | 2.74 |
